# Feasibility of Malaria Diagnosis and Management in Burkina Faso, Nigeria, and Uganda: A Community-Based Observational Study

**DOI:** 10.1093/cid/ciw622

**Published:** 2016-12-06

**Authors:** IkeOluwapo O. Ajayi, Jesca Nsungwa-Sabiiti, Mohamadou Siribié, Catherine O. Falade, Luc Sermé, Andrew Balyeku, Chinenye Afonne, Armande K. Sanou, Vanessa Kabarungi, Frederick O. Oshiname, Zakaria Gansane, Josephine Kyaligonza, Ayodele S. Jegede, Alfred B. Tiono, Sodiomon B. Sirima, Amidou Diarra, Oyindamola B. Yusuf, Florence Fouque, Joëlle Castellani, Max Petzold, Jan Singlovic, Melba Gomes

**Affiliations:** 1 Department of Epidemiology and Medical Statistics; 2 Department of Pharmacology and Therapeutics; 3 Epidemiology and Biostatistics Research Unit, Institute of Advance Medical Research and Training (IMARAT); 4 Department of Health Promotion and Education, Faculty of Public Health, College of Medicine; 5 Department of Sociology, Faculty of Social Sciences, IMARAT, University of Ibadan, Nigeria; 6 Child Health Division, Ministry of Health, Kampala, Uganda; 7 Groupe de Recherche Action en Santé, Ouagadougou, Burkina Faso; 8 UNICEF/UNDP/World Bank/WHO Special Programme for Research & Training in Tropical Diseases, World Health Organization, Geneva, Switzerland; 9 Department of Health Services Research, School for Public Health and Primary Care, Maastricht University, The Netherlands; 10 Centre for Applied Biostatistics, Occupational and Environmental Medicine, Sahlgrenska Academy, University of Gothenburg, Sweden

**Keywords:** malaria treatment access, Africa, prereferral treatment, artemisinin combination treatment, rapid diagnostic tests

## Abstract

***Background.*** Malaria-endemic countries are encouraged to increase, expedite, and standardize care based on parasite diagnosis and treat confirmed malaria using oral artemisinin-based combination therapy (ACT) or rectal artesunate plus referral when patients are unable to take oral medication.

***Methods.*** In 172 villages in 3 African countries, trained community health workers (CHWs) assessed and diagnosed children aged between 6 months and 6 years using rapid histidine-rich protein 2 (HRP2)–based diagnostic tests (RDTs). Patients coming for care who could take oral medication were treated with ACTs, and those who could not were treated with rectal artesunate and referred to hospital. The full combined intervention package lasted 12 months. Changes in access and speed of care and clinical course were determined through 1746 random household interviews before and 3199 during the intervention.

***Results.*** A total of 15 932 children were assessed: 6394 in Burkina Faso, 2148 in Nigeria, and 7390 in Uganda. Most children assessed (97.3% [15 495/15 932]) were febrile and most febrile cases (82.1% [12 725/15 495]) tested were RDT positive. Almost half of afebrile episodes (47.6% [204/429]) were RDT positive. Children eligible for rectal artesunate contributed 1.1% of episodes. The odds of using CHWs as the first point of care doubled (odds ratio [OR], 2.15; 95% confidence interval [CI], 1.9–2.4; *P* < .0001). RDT use changed from 3.2% to 72.9% (OR, 80.8; 95% CI, 51.2–127.3; *P* < .0001). The mean duration of uncomplicated episodes reduced from 3.69 ± 2.06 days to 3.47 ± 1.61 days, Degrees of freedom (df) = 2960, Student's t (t) = 3.2 (*P* = .0014), and mean duration of severe episodes reduced from 4.24 ± 2.26 days to 3.7 ± 1.57 days, df = 749, t = 3.8, *P* = .0001. There was a reduction in children with danger signs from 24.7% before to 18.1% during the intervention (OR, 0.68; 95% CI, .59–.78; *P* < .0001).

***Conclusions.*** Provision of diagnosis and treatment via trained CHWs increases access to diagnosis and treatment, shortens clinical episode duration, and reduces the number of severe cases. This approach, recommended by the World Health Organization, improves malaria case management.

***Clinical Trials Registration.*** ISRCTN13858170.

*Plasmodium falciparum* malaria now causes 214 million cases and 438 000 deaths, mainly in young African children [1]. Parasitological diagnosis through microscopy or rapid diagnostic tests (RDTs) is recommended so that only patients with confirmed malaria are treated with artemisinin-based combination therapies (ACTs) [2], thus reducing ACT wastage and prompting timely management of other infections with similar symptoms [3]. RDTs are relatively easy to use. For severe malaria, transfer to hospital for parenteral artesunate treatment, or rectal artesunate treatment before referral, is beneficial if the journey to hospital is likely to take several hours [2, 4].

Effective malaria management requires that all components be in place—trained health workers (community and facility), provider adherence to treatment guidelines, commodity availability, and patient access and adherence to treatment and referral advice. African malaria-endemic countries have improved parasitological diagnosis from 36% of suspected malaria cases tested in 2005 to 65% in 2014 [5]. The percentage of ACT coverage in 2014 for patients with *P. falciparum* malaria is reported to be 100% from 2012 levels of 96% in Burkina Faso, 36% in Nigeria, and 66% in Uganda, but this increase is primarily due to RDT use in the public sector [1]. In many rural malaria-endemic areas of Africa, children are unable to access effective diagnosis or malaria treatment because public facilities are distant. Therefore, the target of ACT use on the basis of parasitological diagnosis is not met often because children go preferentially to the private sector, where treatment is more readily available but reliable diagnosis is not [1]. Over-the-counter drugs are the first choice for two-thirds of suspected malaria cases in Uganda [6], with comparable proportions elsewhere [7]. Thus, the organization of health systems to facilitate access to reliable diagnosis and prompt treatment continues to be challenging in most low-resource African settings.

We report on research undertaken to understand what it takes to improve coverage for 3 countries (Burkina Faso, Nigeria, and Uganda) chosen because they are in the top 10 countries that contribute 80% of global malaria cases, and in the top 15 that contribute 78% of malaria deaths [1]. Our hypothesis was that prompt, community-based access to RDT diagnosis and treatment would expand and expedite access, shorten episode duration, and reduce private costs of caring for the child. In addition, prompt treatment should reduce severe malaria and the public costs of managing episodes that have evolved to severe malaria and require admission, and expedite management of infections that were erroneously assumed to be malaria.

## METHODS

### Study Sites and Health Care

The study was carried out in rural areas of Burkina Faso (health area of Sidéradougou, Health District of Mangodara), Nigeria (Ona-Ara local government area), and Uganda (Sheema and Kayunga districts).

In Burkina Faso, patients seek care from CHWs, traditional healers, faith homes, drug shops/drug hawkers, dispensaries, and health centers (“centre médical”). For this study, 45 villages were chosen in the health area of Sidéradougou, Mangodara District; CHWs refer very sick patients to the next level of care, “Centre de Santé et de promotion sociale” (managed by a nurse), which refers patients to a health center (managed by a medical doctor), and from there to the regional hospital. There is only 1 inpatient health center in the district. Care given at the Centre de Santé, health center, or hospital is fee-based. A consultation requires approximately 200 West African CFA Franc (XOF) (US Dollars [USD] $.33), and admitted patients pay for their bed.

In Nigeria, 33 study villages were selected for this study. A similar pattern of care to Burkina Faso is observed—use of traditional healers, traditional birth attendants, CHWs, health centers, maternity wards, and private clinics (usually managed by a nurse with admissions shorter than 24 hours) as well as shops/patent medicine sellers/drug hawkers where quinine, antibiotics, and antimalarials without prescription are available. In the study sites, there are 8 health centers managed by facility-based CHWs and community health extension workers. The centers are open at all hours if there are admissions; in the absence of admissions they close around 4–6 pm. The policy is that consultations, drugs, and bed costs at public facilities are provided without charge for young children aged <5 years, although caregivers pay for registration cards and injections.

Uganda has a similar variety of traditional healers, faith homes, drug shops, and private clinics and several grades of health centers. The 84 study villages in Uganda (of which 32 participated in the preintervention study) have 2 grade IV admitting health centers, 1 in each district, with no district hospital. Medical care is provided without charge, but patients pay for hospital registration and medications.

### Commodities

Before the intervention, no RDTs, ACTs, or rectal artesunate were routinely available through CHWs in any of the communities. Nationally approved RDTs (Malaria histidine-rich protein 2 [HRP2] [*Pf*], CareStart™ in Burkina Faso, Bioline SD in Nigeria and Uganda) and ACTs (Coartem) supplied in 2 prepacked weight- and age-specific doses were procured by the World Health Organization (WHO) for the study in Burkina Faso and Nigeria and through the Global Fund in Uganda. In Nigeria, the Federal Ministry of Health also provided RDT kits and the Primary Health Centre provided some Coartem. Rectal artesunate was provided for all countries by the WHO TDR (Knoll/Catalent/Scanpharm) in October 2014. Treatment with rectal artesunate was covered by insurance.

### Intervention: Identification and Training of CHWs

CHWs were either not available (Uganda) or barely utilized (Burkina Faso and Nigeria) prior to the study; thus, the study began by supporting the identification and training of CHWs according to national guidelines in all 3 countries. Communities were visited, the study was explained, and community agreement was obtained to participate. If communities agreed, they were informed that existing CHWs would be selected and trained for the intervention; if not, available community members to be trained as CHWs would be identified using local criteria (residence, availability, literacy, and acceptability) before training. In the intervention, each CHW would be responsible for his or her own catchment area.

Once CHWs were identified, training was carried out by health staff (Burkina Faso and Uganda) or the investigative team (Nigeria) in sessions that covered theory and practice [8]. Training included diagnosis and recognition of uncomplicated and severe illness with danger signs, counting respiratory rate, diagnosis using RDTs (preparing, reading, recording results), and treatment dosage by age. For every RDT-positive case, CHWs were taught to explain RDT results and emphasize the importance of providing the child with complete doses of Coartem tablets. All children who did not improve were to be brought back. CHWs were taught to use symptoms to identify children eligible for rectal artesunate, obtain informed consent before treatment, and explain the importance of proceeding to hospital to complete management. They were taught to document treatment and drugs, periodically reviewed by supervisors. Children treated with rectal artesunate were to be followed up at home for outcome. In Nigeria, CHWs were trained to prepare a thick blood smear for later microscopy in the project laboratory [9].

Training was for 5 days in Uganda and 3 days in Nigeria (with an additional 2 days for severe malaria) and 3 days in Burkina Faso. Training was evaluated through pre- and post-tests [8]. A total of 31, 40, and 164 CHWs passed all certification exams in Burkina Faso, Nigeria, and Uganda, respectively, and were authorized to diagnose and treat uncomplicated and severe malaria, but in Burkina Faso 19 CHWs failed the severe malaria component necessary for use of rectal artesunate, and were only authorized to diagnose and use ACTs. Each CHW was issued with the necessary commodities and other materials at the time of certification.

All countries implemented the combined intervention of RDTs, ACTs, and rectal artesunate via trained CHWs from October 2014 to October 2015; Nigeria began earlier with RDTs and ACTs in December 2013.

### CHW Costs and Logistics

CHWs were not paid in the participating countries in the study. In Burkina Faso, CHWs bought subsidized Coartem and were authorized to retail the same to patients (as per government policy) at 100 XOF (USD $.17) for children up to 36 months and 200 XOF (USD $.33) for children between 37 and 59 months giving them a small profit on each treatment. In Nigeria, CHWs were provided gifts (during festivities) and performance motivation; CHWs’ texts/phone calls to the team regarding specific patients were reimbursed and a stipend was given for team meeting days. Patients were not charged for ACTs in Nigeria and Uganda, and rectal artesunate was provided at no cost to the patient in all countries.

### Supervision

In Nigeria, after study initiation, the study team supervised CHWs with visits for review and correction of case report forms (CRFs)/registers, replenishment of drug supplies, and regular monitoring. In Uganda, the 5 nurses, 2 clinical officers, and 3 other facility staff belonging to the 7 facilities in the study areas were assigned supervisory roles and trained to support and supervise CHW performance, manage the supply chain, and support CHWs to manage referrals. Quarterly meetings were organized with transport allowance for CHWs to review progress. Supervision also occurred during a monthly visit posttraining.

### Involvement of Health Structure Including Primary Facilities and Hospitals

In each country, meetings were held with local health staff informing them about the study, its purposes and procedures, and their roles in the study, especially for referrals to their facility. In Burkina Faso, 16 nurses and 1 physician (from the only facility) were trained; training included supervision of CHWs. There was refresher training on management of severe patients in the community, facility completion of documentation, tracking of adverse events, commodity and supply accountability, and supervision of CHWs. Field laboratory technicians were retrained in microscopy and storing blood slides for subsequent expert rereading. Medical staff and the inventory controller received stipends. In Nigeria, visits were made to 8 primary health centers in the study area to explain the study to nurses, community health extension workers (CHEWs), junior CHEWs, 1 doctor, and 19 local health staff; all including the malaria focal person for the area were trained. The study covered all training costs including transport. In Uganda, the intervention was organized and implemented by the Ministry of Health. Thus, the procedure for identification, training, supervision, monitoring, and evaluation of CHWs was integrated with routine care procedures for scale-up of integrated Community Management of Malaria (iCCM).

### Documentation of Treatment/Quality Control

Special registers for Burkina Faso and Nigeria and routine iCCM registers in Uganda were utilized to document information on sick children seeking treatment. Every patient assessed was registered and data were obtained on date/time of visit, gender, date of birth, symptoms, known allergies, RDT results, and treatment given. In Burkina Faso and Nigeria, RDT cassettes were collected and reread centrally; In Burkina Faso, blood slides of referred cases were read at the referral hospital and reread centrally [10]. In Nigeria, thick blood smears were made from the same blood spot used for RDTs for all cases [9].

### Household Surveys

The impact of the intervention was evaluated through pre- and post-quantitative household surveys to assess impact (changes in treatment-seeking behavior), qualitative surveys that documented acceptability of the program [11], and an economic evaluation of the package [12]. In addition, CHW performance was assessed.

Except for use of French in Burkina Faso, household interviews were in the local languages (Yoruba in Nigeria; Luganda and Lunyankole in Uganda), with forms pretested before use. Trained interviewers were fluent in local languages, experienced in data collection, and with a minimum of secondary school certificate in Nigeria and undergraduate degree in Uganda and Burkina Faso. They were trained together, tested in the language of interview and in CRF completion. Ten interviewers were selected in Burkina Faso and Nigeria and 8 in Uganda for the pre-intervention phase and 10 in Burkina Faso, 13 in Nigeria, and 20 in Uganda for the intervention phase.

Household surveys were carried out before the intervention and once the intervention had begun. A 2-stage sampling process was used to select households for the survey: First, participating villages were randomly selected, and within villages, households were randomly selected. Households whose caregiver was not present during the illness or who refused to give consent were not interviewed. When a household had >1 sick child within the prior 2 weeks, the first child to be sick was chosen in Burkina Faso, all in Nigeria, but the episode of the youngest child was chosen in Uganda. If the caregiver was absent, the interviewer returned to reattempt the interview; a futile second attempt in Burkina Faso and Uganda and third attempt in Nigeria classified the household as lost for interview.

### Sample Size Calculations and Statistical Analyses

In calculating our sample size for household surveys, we assumed that the average number of children aged <5 years per household was 2, except in Uganda where the average was assumed as 1.4. Requiring a precision of ±5% points for the point estimates of proportions, a minimum sample size would be 384 for the worst-case scenario of 50%. Accounting for a design effect of 2 for clustering on household level would give us a sample size of 2 × 384 = 768 interviews before and during the intervention in each country. We achieved interviews with 1856 caretakers in Burkina Faso (514 before, 1342 after), 1560 in Nigeria (775 before, 785 after) and 1529 in Uganda (457 before, 1072 after) with an overall total of 4945 households interviewed. Data were entered into EpiData version 3.1. All statistical analyses were performed using Stata software, version 14 (StataCorp). Simple proportions were used for most analyses, and differences between groups were evaluated by χ^2^ test at *P* < .05.

### Ethical Approval

Ethical approval for conduct of the study was obtained from the WHO Ethics Review Committee and from national ethical review boards (the National Health Research Committee and Oyo State Ministry of Health in Nigeria; the National Ethics Committee for the Research on Health in Burkina Faso; and the National Council for Science and Technology in Uganda). Informed consent was obtained in accordance with the ethical standards set by the different ethics committees.

## RESULTS

**Figure 1.**
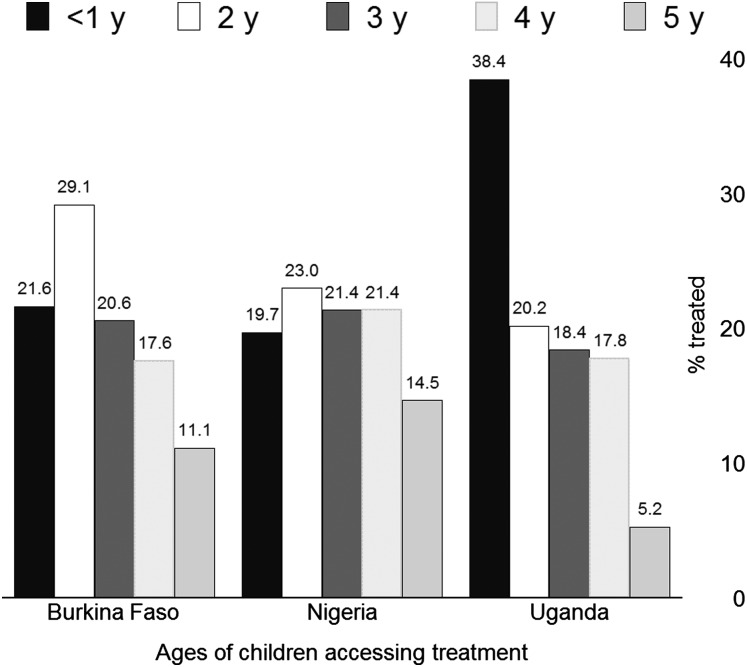
Age-specific access to treatment, by country.

**Figure 2.**
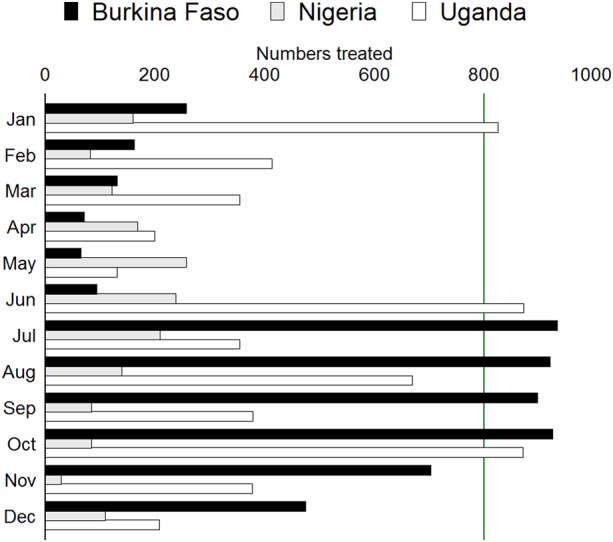
Monthly access to treatment, by country.

A total of 15 932 patients consulted trained CHWs during 22 months in Nigeria and 12 months in Burkina Faso and Uganda. About half (53%) of the children seeking care were aged between 6 and 24 months, 71% being <36 months of age (Figure 1). The number of children coming for care was 6394 in Burkina Faso, 2148 in Nigeria, and 7390 in Uganda (Figure 2).

The vast majority of episodes (97.3% [15 495/15 932]) for which patients came for care were febrile, and the majority (82.1% [12 725/15 495]) were diagnosed as parasitologically positive using RDTs (Table 1). However, an important number of children (2.7%; 423 uncomplicated, and 6 unable to take oral medication) were brought in with no history of fever or temperature and 47.6% (204/429) were RDT positive, most uncomplicated cases without any other symptoms (vomiting, diarrhea, cough, or fast breathing).

**Table:** Table 1. Overview of the Number of Patients Who Came for Care During the Intervention: Uncomplicated and Severe Patients

	Burkina		Nigeria		Uganda			
	Total	%	Total	%	Total	%	Total	%
**Uncomplicated patients: Oral treatment**								
Fever Positive								
RDT positive+	**5191**	83.5	**1630**	76.8	**5785**	78.5	**12606**	80.1
ACTs-Coartem	5179		1629		5619		12427	
Other Treatment^a^					53		53	
No/Unknown Treatment	12		1		113		126	
RDT negative-	**819**	13.2	**478**	22.5	**1337**	18.1	**2634**	16.7
ACTs-Coartem	7		16		4		27	
Other Treatment^a^					598		598	
No/Unknown Treatment	812		462		735		2009	
RDT not done	**15**	0.2	**0**	…	**56**	0.8	**71**	0.5
ACTs-Coartem	2				4		6	
Other Treatment^a^					37		37	
No Treatment	13				15		28	
RDT not known	**1**	0.02	**0**	…	**10**	0.1	**11**	0.07
ACTs-Coartem	1				6		7	
No Treatment^a^					1		1	
Unknown Treatment					3		3	
Total Febrile	**6026**	96.9	**2108**	99.3	**7188**	97.6	**15322**	97.3
Fever Negative								
RDT positive+	**180**	2.9	**2**	0.1	**21**	0.3	**203**	1.3
ACTs-Coartem	178		2		20		200	
No/Other Treatment^a^	2				1		3	
RDT negative-	**49**	0.8	**13**	0.6	**108**	1.5	**170**	1.1
ACTs-Coartem	1		1				2	
Other Treatment^a^			0		63		63	
No/Unknown Treatment	48		12		45		105	
RDT not done	**0**	…	**0**	…	**45**	0.6	**45**	0.3
ACTs-Coartem					1		1	
No/Other Treatment^a^					44		44	
RDT not known					**5**	0.1	**5**	0.03
Total Afebrile	**229**	3.7	**15**	0.7	**179**	2.4	**423**	2.7
Total uncomplicated cases	**6255**		**2123**		**7375^b^**		**15753^b^**	
**Severe patients: All with danger signs preventing oral treatment, eligible for rectal artesunate^c^**								
Fever positive								
RDT positive+	81	58.3	25	100	13	86.7	119	66.5
RDT negative-	45	32.4			2	13.3	47	26.3
RDT not done	7	5.0					7	3.9
Fever negative								
RDT positive+	1	0.7					1	0.6
RDT negative-	5	3.6					5	2.8
Total severe cases	**139**		**25**		**15**		**179**	
Grand Total assessed/ treated	**6394**		**2148**		**7390^b^**		**15932^b^**	

Subtotals are in bold.

Abbreviations: ACT, artemisinin-based combination therapy; RDT, rapid diagnostic test; WHO, World Health Organization.

^a^ Other treatment included antibiotics, analgesics, vitamins, tonics.

^b^ Including 8 children, all in Uganda, who had no information on RDT use or fever, reportedly uncomplicated.

^c^ Danger signs defined as in WHO Integrated Management of Childhood Illness (IMCI) Treatment Guidelines: repeated vomiting, fast breathing, convulsions, weakness/lethargy, coma/altered consciousness; all *Nil-per os.*

Most (98.6% [12 427/12 606]) febrile and afebrile (98.5% [200/203]) uncomplicated malaria cases were treated with an ACT (Table 1). Very few RDT-negative uncomplicated episodes (0.2% [29/12 656]) were treated with ACTs [13]. In a small number of patients (0.87% [139/15 932]), RDT diagnosis either was not done or results were not documented.

Children who could not take oral medications, eligible for rectal artesunate, contributed a very small fraction of episodes: 1.1% (179/15 932), and almost all febrile (Table 1). More than two-thirds of RDT-tested children with danger signs (69.7% [120/172]) were positive, the remaining were RDT negative, except for 7 febrile patients where the RDT was not done. There were 30 children with rapid breathing among those with danger signs eligible for rectal artesunate.

Table 2 provides more details of the children who were treated with rectal artesunate, the majority from Burkina Faso. Most children were aged <24 months. Altogether, 24% of children had a combination of altered consciousness/coma or convulsions, and 13.4% had altered consciousness/coma. The mean delay between onset of symptoms and treatment with rectal artesunate by a CHW ranged from a few minutes to 5 days (median, 22.4 hours overall).

**Table 2.** Overview of Patients Receiving Rectal Artesunate Treatment

Rectal Artesunate Treatment	Burkina Faso	Nigeria	Uganda	Total
Total No. treated	139	25	15	179
Child's age				
<12 mo	32 (23.0)	3 (12.0)	2 (13.3)	37 (20.7)
12–24 mo	58 (41.7)	6 (24.0)	5 (33.3)	69 (38.5)
>24–36 mo	27 (19.4)	7 (28.0)	2 (13.3)	36 (20.1)
>36–48 mo	13 (9.4)	7 (28.0)	3 (20.0)	23 (12.8)
>48–59 mo	9 (6.5)	2 (8.0)	3 (20.0)	14 (7.8)
Symptoms				
Prostrated and unable to take oral medication	104 (74.8)	3 (12.0)	5 (33.3)	112 (62.6)
Altered consciousness + convulsions	14 (10.1)	19 (76.0)	10 (66.7)	43 (24.0)
Altered consciousness or coma	21 (15.1)	3 (12.0)	…	24 (13.4)
Compliance with referral advice				
No compliance	3 (2.2)	5 (20.0)	8 (53.3)	16 (8.9)
Arrived at facility	134 (96.4)	18 (72.0)	3 (20.0)	155 (86.6)
Drug shop	…	2 (8.0)	1 (6.7)	3 (1.7)
Traditional healer	2 (1.4)	…	1 (6.7)	3 (1.7)
Went elsewhere	…	…	2 (13.3)	2 (1.1)
Median delay from symptom onset to treatment, h	22.9	18.5	24	22.4
No. of patients with information (IQR)	135 (8.2–47.3)	25 (4–30.8)	10 (6.5–24)	170 (8.1–46.9)
Median interval from treatment to facility arrival, h (IQR)^a^	2.1^a^ (1.2–3.4)^a^	1 (0.8–1.3)	2.5 (1.5–7.3)	2.1^a^ (1–3.3)^a^
No. of patients with information^a^	134	18	4	156
Treatment at hospital: RDT-positive cases				
None	4 (4.9)	10 (40.0)	10 (76.9)	24 (20.0)
IV quinine	…	1 (4.0)	1 (7.7)	2 (1.7)
ACT + IV quinine	10 (12.2)	…	…	10 (8.3)
IV antibiotic + antimalarial	8 (9.8)	3 (12.0)	2 (15.4)	13 (10.8)
Transfusion	…	3 (12.0)	…	3 (2.5)
Oral quinine	…	1 (4.0)	…	1 (0.8)
Oral antibiotic + antimalarial	60 (73.2)	7 (28.0)	…	67 (55.8)
Total RDT positive	82	25	13	120
Treatment at hospital: RDT-negative cases				
No treatment	2 (4.0)	…	2 (100.0)	4 (7.7)
ACT + IV quinine	2	…	…	2 (3.8)
IV antibiotic + antimalarial	2	…	…	2 (3.8)
Oral antibiotic + antimalarial	44	…	…	44 (84.6)
Total RDT negative	50	…	2	52
Outcome at follow-up				
Alive/well at interview	134 (96.4)	21 (84.0)	14 (93.3)	169 (94.4)
Alive/hospitalized	…	2 (8.0)	…	2 (1.1)
Alive/not present at interview	…	…	1 (6.7)	1 (0.6)
Died	5 (3.6)	2 (8.0)	…	7 (3.9)

Data are presented as No. (%) unless otherwise indicated.

Abbreviations: ACT, artemisinin-based combination therapy; IQR, interquartile range; IV, intravenous; RDT, rapid diagnostic test.

^a^ Excluding 1 Burkina Faso patient who reported arrival at the facility after 22 days.

Compliance with advice to proceed to the nearest health facility among those referred varied from 20% (3/15) among fewer patients in Uganda to 96.4% (134/139) in a larger series in Burkina Faso. The delay between treatment and arrival at the referral facility ranged from 15 minutes to 31 hours. The median interval was 1 hour in Nigeria (with transport facilitated by the study team), 2.1 hours in Burkina Faso, and 2.5 hours in Uganda. Treatment at the facility for RDT-positive patients was mainly oral antibiotics and antimalarials. CHWs followed up most children at their home within 3 days of treatment; at follow-up, there were no sequelae reported or seen; 2 guardians reported weakness after the episode (1 in Burkina Faso and 1 in Uganda), but the child had recovered by the second follow-up.

Seven children treated with rectal artesunate died (5 in Burkina Faso and 2 in Nigeria). Three Burkina Faso fatalities were RDT positive, all treated with rectal artesunate, and went to hospital; the hospital discharge diagnosis was severe malaria, although microscopy at hospital was negative. The remaining 2 Burkina Faso children were RDT negative, 1 with a diagnosis of acute meningitis/bacterial gastroenteritis and severe malnutrition (confirmed microscopy negative at the hospital); the other RDT-negative patient did not arrive at hospital. Two children died in Nigeria: both were RDT positive; 1 had severe anemia, high parasitemia and died 2 days after admission at the hospital while awaiting transfusion; the second child died 30 minutes posttreatment with rectal artesunate, en route to hospital.

Community-based diagnosis and treatment was expected to increase use of CHWs, reduce time to malaria diagnosis and treatment, shorten episode duration, and prevent evolution to severe malaria. Household interviews with randomly chosen caretakers of recently ill children were intended to capture changes in treatment-seeking behavior associated with the intervention. Table 3 provides baseline characteristics of the households interviewed. There was no apparent difference with regard to the sick child's gender or age, or the caregiver's age, education, or socioeconomic status. The sole difference is caregiver's gender before vs during the intervention in Burkina Faso, where significantly more males responded to questions about the episode before the intervention.

**Table 3.** Characteristics of Households Randomly Chosen for Interview: Before Versus During Intervention

	Before Intervention				During Intervention			
	Burkina Faso	Nigeria	Uganda	Total	Burkina Faso	Nigeria	Uganda	Total
Total No. of caregivers interviewed	514 (29.4)	775 (44.4)	457 (26.2)	1746 (100.0)	1342 (42.0)	785 (24.5)	1072 (33.5)	3199 (100.0)
Child's gender								
Male	275 (53.5)	400 (51.6)	236 (51.6)	911 (52.2)	724 (53.9)	355 (45.2)	545 (50.8)	1624 (50.8)
Female	239 (46.5)	375 (48.4)	221 (48.4)	835 (47.8)	617 (46.0)	430 (54.8)	506 (47.2)	1553 (48.5)
Missing information	0 (0.0)	0 (0.0)	0 (0.0)	0 (0.0)	1 (0.1)	0 (0.0)	21 (2.0)	22 (0.7)
Child's age								
<6 mo	18 (3.5)	7 (0.9)	15 (3.3)	40 (2.3)	1 (0.1)	3 (0.4)	61 (5.7)	65 (2.0)
6–11 mo	56 (10.9)	81 (10.5)	59 (12.9)	196 (11.2)	176 (13.1)	65 (8.3)	127 (11.8)	368 (11.5)
12–23 mo	135 (26.3)	180 (23.2)	116 (25.4)	431 (24.7)	409 (30.5)	177 (22.5)	261 (24.3)	847 (26.5)
24–35 mo	118 (23.0)	180 (23.2)	92 (20.1)	390 (22.3)	319 (23.8)	194 (24.7)	238 (22.2)	751 (23.5)
36–47 mo	89 (17.3)	179 (23.1)	92 (20.1)	360 (20.6)	231 (17.2)	171 (21.8)	188 (17.5)	590 (18.4)
48–59 mo	89 (17.3)	147 (19.0)	83 (18.2)	319 (18.3)	206 (15.4)	175 (22.3)	165 (15.4)	546 (17.1)
>59 mo	0 (0.0)	0 (0.0)	0 (0.0)	0 (0.0)	0 (0.0)	0 (0.0)	14 (1.3)	14 (0.4)
Missing information	9 (1.8)	1 (0.1)	0 (0.0)	10 (0.6)	0 (0.0)	0 (0.0)	18 (1.7)	18 (0.6)
Caregiver's gender								
Male	220 (42.8)	62 (8.0)	64 (14.0)	346 (19.8)	29 (2.2)	17 (2.2)	77 (7.2)	123 (3.8)
Female	294 (57.2)	713 (92.0)	393 (86.0)	1400 (80.2)	1313 (97.8)	768 (97.8)	992 (92.5)	3073 (96.1)
Missing information	…	…	…	…	…	…	3 (0.3)	3 (0.1)
Caregiver's age								
15–24 y	82 (16.0)	123 (15.9)	92 (20.1)	297 (17.0)	325 (24.2)	130 (16.6)	277 (25.8)	732 (22.9)
25–35 y	225 (43.8)	461 (59.5)	226 (49.5)	912 (52.2)	786 (58.6)	452 (57.6)	498 (46.5)	1736 (54.3)
36–50 y	153 (29.8)	137 (17.7)	100 (21.9)	390 (22.3)	182 (13.6)	168 (21.4)	226 (21.1)	576 (18.0)
>50 y	34 (6.6)	32 (4.1)	37 (8.1)	103 (5.9)	11 (0.8)	32 (4.1)	47 (4.4)	90 (2.8)
Unknown	20 (3.9)	22 (2.8)	2 (0.4)	44 (2.5)	38 (2.8)	3 (0.4)	24 (2.2)	65 (2.0)
Caregiver's education								
No education	408 (79.4)	189 (24.4)	55 (12.0)	652 (37.3)	1202 (89.6)	171 (21.8)	120 (11.2)	1493 (46.7)
≤7 y	43 (8.4)	312 (40.3)	281 (61.5)	636 (36.4)	106 (7.9)	279 (35.5)	652 (60.8)	1037 (32.4)
>7 y	21 (4.1)	273 (35.2)	121 (26.5)	415 (23.8)	26 (1.9)	335 (42.7)	280 (26.1)	641 (20.0)
Missing information	42 (8.2)	1 (0.1)	0 (0.0)	43 (2.5)	8 (0.6)	0 (0.0)	20 (1.9)	28 (0.9)
Occupation^a^								
Unemployed	11 (2.1)	27 (3.5)	9 (2.0)	47 (2.7)	35 (2.6)	1 (0.1)	31 (2.9)	67 (2.1)
Agriculture only	470 (91.4)	270 (34.8)	370 (81.0)	1110 (63.5)	687 (51.2)	190 (24.2)	794 (74.1)	1671 (52.2)
Employed only	2 (0.4)	24 (3.1)	5 (1.1)	31 (1.8)	10 (0.7)	31 (3.9)	21 (2.0)	62 (1.9)
Self-employed only	21 (4.1)	278 (35.9)	12 (2.6)	311 (17.8)	96 (7.2)	342 (43.6)	83 (7.7)	521 (16.3)
Employed + agriculture	…	3 (0.4)	12 (2.6)	15 (0.9)	…	9 (1.1)	24 (2.2)	33 (1.0)
Self-employed + agriculture	9 (1.8)	137 (17.7)	47 (10.3)	193 (11.1)	514 (38.3)	123 (15.7)	103 (9.6)	740 (23.1)
Other	…	34 (4.4)	2 (0.4)	36 (2.1)	…	89 (11.3)	3 (0.3)	92 (2.9)

Data are presented as No. (%).

^a^ Missing information for 1 in Burkina, 2 in Nigeria before intervention; missing information for 13 in Uganda during intervention.

Table 4 outlines the impact of the intervention on treatment-seeking behavior, access, and use of CHWs as the first point of care, diagnosis, treatment, and speed of care. The survey data before the intervention testify to either the very low use of CHWs as the first point of care or the absence of and low use of CHWs. Post-intervention, there was a significant increase in using CHWs as first point of care, from 31.1% (543/1746) to 49.2% (1575/3199), with a doubling in the odds ratio (OR, 2.15; 95% confidence interval [CI], 1.9–2.4; *P* < .0001). The use of trained CHWs increased in general (not only as a first resort), from 35.5% (620/1746) before to 50.2% (1606/3199) during the intervention, thus increasing CHW use by 83% (OR, 1.83; 95% CI, 1.62–2.06; *P* < .0001).

**Table 4.** Effect of Intervention on Diagnosis and Treatment of Sick Children

Total No. of Parents Interviewed	Before Intervention				During Intervention			
	Burkina Faso	Nigeria	Uganda	Total	Burkina Faso	Nigeria	Uganda	Total
	514 (29.4)	775 (44.4)	457 (26.2)	1746 (100.0)	1342 (42.0)	785 (24.5)	1072 (33.5)	3199 (100.0)
Did the caregiver go to the CHW as first provider?								
No	409 (79.6)	337 (43.5)	457 (100)	1203 (68.9)	741 (55.2)	260 (33.1)	623 (58.1)	1624 (50.8)
Yes	105 (20.4)	438 (56.5)	…	**543** **(31.1)**	601 (44.8)	525 (66.9)	449 (41.9)	**1575** **(49.2)**
How many went to trained health workers?								
Trained CHWs/health workers	105 (20.4)	515 (66.5)	…	**620** **(35.5)**	603 (44.9)	534 (68.0)	469 (43.8)	**1606** **(50.2)**
Other providers	409 (79.6)	260 (33.5)	457 (100)	1126 (64.5)	739 (55.1)	251 (32.0)	603 (56.3)	1593 (49.8)
Was an RDT diagnosis done?								
No	95 (90.5)	494 (95.9)	…	589 (95.0)	32 (5.3)	239 (44.8)	148 (31.6)	419 (26.1)
Yes	2 (1.9)	18^a^ (3.5)	…	**20** **(3.2)**	567 (94.0)	293 (54.9)	311 (66.3)	**1171** **(72.9)**
Missing	8 (7.6)	3 (0.6)	…	11 (1.8)	4 (0.7)	2 (0.4)	10 (2.1)	16 (1.0)
Were ACTs given (via any provider)?								
No	345 (67.1)	686 (88.5)	205 (44.9)	1236 (70.8)	580 (43.2)	443 (56.4)	439 (41.0)	1462 (45.7)
Yes	169 (32.9)	89 (11.5)	252 (55.1)	**510** **(29.2)**	762 (56.8)	342 (43.6)	633 (59.0)	**1737** **(54.3)**
Was the child treated within 24 h?								
Yes	224 (43.6)	482 (62.2)	335 (73.3)	1041 (59.6)	675 (50.3)	465 (59.2)	844 (78.7)	1984 (62.0)
No	67 (13.0)	64 (8.3)	84 (18.4)	215 (12.3)	171 (12.7)	114 (14.5)	116 (10.8)	401 (12.5)
No treatment /other treatment	223 (43.4)	229 (29.5)	38 (8.3)	490 (28.1)	496 (37.0)	206 (26.2)	112 (10.4)	814 (25.4)

Data are presented as No. (%) unless otherwise indicated. Values in bold compare behavior before vs during the intervention and indicate significance at *P* < .0001.

Abbreviations: ACT, artemisinin-based combination therapy; CHW, community health worker; RDT, rapid diagnostic test.

^a^ This total includes 6 RDT diagnoses made by drug-shop owners.

Table 4 also shows that the population of sick children diagnosed with RDTs changed from 3.2% (20/620) before the intervention to 72.9% after initiation (1171/1606) (OR, 80.8; 95% CI, 51.2–127.3; *P* < .0001). ACT treatment through any provider increased from 29.2% (510/1746) to 54.3% (17 37/3199) (OR, 2.87; 95% CI, 2.54–3.26; *P* < .0001).

Table 5 shows the differences in speed of clinical resolution of the episode and clinical outcomes for the 1746 cases before and 3199 during the intervention. Some 20%–30% of episodes had not resolved at the time of the interviews. For children whose episodes had resolved before interview, the mean duration of uncomplicated episodes decreased from 3.69 ± 2.06 days to 3.47 ± 1.61 days, degrees of freedom (df) = 2960, Student's t (t) = 3.2 (*P* = .0014), and mean duration of severe episodes decreased from 4.24 ± 2.26 days to 3.7 ± 1.57 days, df = 749, t = 3.8 (*P* = .0001).

**Table 5.** Impact of Intervention on Clinical Outcomes

Total No. of Caretakers Interviewed	Before Intervention				During Intervention			
	Burkina Faso	Nigeria	Uganda	Total	Burkina Faso	Nigeria	Uganda	Total
	514 (29.4)	775 (44.4)	457 (26.2)	1746 (100.0)	1342 (42.0)	785 (24.5)	1072 (33.5)	3199 (100.0)
Mean duration of episode, completed episodes^a^								
Uncomplicated episodes, mean (SD)	288 (3.45 [1.60])	492 (3.17 [1.85])	258 (4.94 [2.34])	**1038 (3.69 [2.06])**	880 (3.19 [1.11])	529 (3.33 [1.73])	515 (4.11 [1.99])	**1924 (3.47 [1.61])**
Severe episodes, mean (SD)	52 (3.69 [1.77])	156 (3.67 [1.88])	146 (5.03 [2.55])	**354 (4.24 [2.26])**	144 (3.52 [1.26])	121 (3.67 [1.71])	132 (3.93 [1.72])	**397 (3.70 [1.57])**
Episode outcome								
Alive and well	344 (66.9)	648 (83.6)	403 (88.2)	1395 (79.9)	1055 (78.6)	650 (82.8)	658 (61.4)	2363 (73.9)
Died	2 (0.4)	…	1 (0.2)	3 (0.2)	1 (0.1)	…	…	1 (0.0)
Still sick	168 (32.7)	127 (16.4)	53 (11.6)	348 (19.9)	280 (20.9)	135 (17.2)	411 (38.3)	826 (25.8)
Missing	…	…	…	…	6 (0.4)	0 (0.0)	3 (0.3)	9 (0.3)

Data are presented as No. (%) unless otherwise indicated. Values in bold compare behavior before vs during the intervention and indicate significance at *P* ≤ .01.

Abbreviation: SD, standard deviation.

^a^ Total excludes 6 patients before and 54 patients after the intervention with resolved episodes, but missing values for episode duration.

We also classified children who had danger signs or not, thus separating the proportion of children with and without severe symptoms before vs during the intervention (Table 6). There was a 32% reduction in children classified as having danger signs, from 24.7% before to 18.1% during the intervention (OR, 0.68; 95% CI, .59–.78; *P* < .0001).

**Table 6.** Effect of Intervention on Symptoms of Children Brought to the Community Health Worker for Assessment

Total No. of Parents Interviewed	Before Intervention				During Intervention			
	Burkina Faso	Nigeria	Uganda	Total	Burkina Faso	Nigeria	Uganda	Total
	514 (29.4)	775 (44.4)	457 (26.2)	1746 (100.0)	1342 (42.0)	785 (24.5)	1072 (33.5)	3199 (100.0)
Symptoms of child^a^								
Fever	511 (99.4)	769 (99.2)	405 (88.6)	1685 (96.5)	1325 (98.7)	784 (99.9)	1027 (95.8)	3136 (98.0)
Diarrhea	119 (23.2)	104 (13.4)	60 (13.1)	283 (16.1)	340 (25.3)	45 (5.7)	149 (13.9)	534 (16.7)
Cough	100 (19.5)	165 (21.3)	197 (43.1)	462 (26.5)	110 (8.2)	83 (10.6)	553 (51.6)	746 (23.3)
Anorexia	4 (0.8)	72 (9.3)	3 (0.7)	79 (4.5)	2 (0.1)	57 (7.3)	4 (0.4)	63 (2.0)
Headache	40 (7.8)	15 (1.9)	0 (0.0)	55 (3.2)	93 (6.9)	18 (2.3)	43 (4.0)	154 (4.8)
Stomach pain	37 (7.2)	8 (1.0)	0 (0.0)	45 (2.6)	77 (5.7)	5 (0.6)	26 (2.4)	108 (3.4)
Pallor	3 (0.6)	21 (2.7)	0 (0.0)	24 (1.4)	2 (0.1)	8 (1.0)	0 (0.0)	10 (0.3)
Leg/body swelling	4 (0.8)	1 (0.1)	0 (0.0)	5 (0.3)	4 (0.3)	0 (0.0)	7 (0.7)	11 (0.3)
Unable to eat/drink/suck	91 (17.7)	109 (14.1)	46 (10.1)	246 (14.1)	223 (16.6)	74 (9.4)	124 (11.6)	421 (13.2)
Mild vomiting	198 (38.5)	107 (13.8)	111 (24.3)	416 (23.8)	574 (42.8)	113 (14.4)	192 (17.9)	879 (27.5)
Repeated vomiting	21 (4.1)	33 (4.3)	17 (3.7)	71 (4.1)	85 (6.3)	31 (3.9)	14 (1.3)	130 (4.1)
Fast breathing	9 (1.8)	31 (4.0)	10 (2.2)	50 (2.9)	2 (0.1)	4 (0.5)	36 (3.4)	42 (1.3)
Convulsion	28 (5.4)	41 (5.3)	78 (17.1)	147 (8.4)	78 (5.8)	40 (5.1)	47 (4.4)	165 (5.2)
Weakness/lethargy	22 (4.3)	116 (15.0)	78 (17.1)	216 (12.4)	59 (4.4)	92 (11.7)	121 (11.3)	272 (8.5)
Coma/altered consciousness	2 (0.4)	3 (0.4)	1 (0.2)	6 (0.3)	5 (0.4)	2 (0.3)	2 (0.2)	9 (0.3)
Bulging fontanel	1 (0.2)	5 (0.6)	1 (0.2)	7 (0.4)	2 (0.1)	0 (0.0)	0 (0.0)	2 (0.1)
Other symptoms	75 (14.6)	168 (21.7)	102 (22.3)	345 (19.8)	244 (18.2)	115 (14.6)	394 (36.8)	753 (23.5)
Other infections	12 (2.3)	18 (2.3)	0 (0.0)	30 (1.7)	23 (1.7)	12 (1.5)	36 (3.4)	71 (2.2)
Danger signs^b^								
No	437 (85.0)	588 (75.9)	290 (63.5)	1315 (75.3)	1129 (84.1)	631 (80.4)	859 (80.1)	2619 (81.9)
Yes	77 (15.0)	187 (24.1)	167 (36.5)	**431 (24.7)**	213 (15.9)	154 (19.6)	213 (19.9)	**580 (18.1)**

Data are presented as No. (%). Values in bold reflect differences in symptoms before vs during the intervention at significance of *P* < .0001.

^a^ Children had >1 symptom; consequently, the symptoms do not equal the number of children.

^b^ Symptoms categorized as being accompanied or not accompanied by an Integrated Management of Childhood Illness danger sign (repeated vomiting, fast breathing, convulsions, weakness/lethargy, coma/altered consciousness, bulging fontanel); children with any danger sign were placed in the “yes” category.

## DISCUSSION

Our results provide strong evidence that providing training, diagnostics, and malaria medicines to CHWs increases coverage, expedites diagnosis and treatment, and shortens the clinical course of both uncomplicated and severe malaria episodes. Severe malaria reflects delays in effective antimalarial treatment. Speed of recovery is important for the child and the family who bear the costs [12]. Reassuringly, there were very few severe malaria cases, even fewer malaria-negative patients treated with an ACT, and deaths were rare. These findings are based on about 16 000 patients assessed, diagnosed, treated, referred, and followed up, and about 5000 households visited to probe behavior changes associated with the intervention.

WHO recommends that malaria case management be based on parasite diagnosis in all cases [1]. This very large study of the treatment of uncomplicated and severe malaria combining RDTs, ACTs, and rectal artesunate shows very high (82.1% [12 725/15 495]) RDT-positive rates among febrile cases. HRP2 tests may well overestimate positivity [14], but even assuming there is some overestimation, it is apparent that declines in transmission documented elsewhere in Africa [15] have not yet reached these villages in 3 countries within the top 15 for malaria prevalence and deaths. The extent to which malaria can be reduced in such high-transmission areas of Africa remains to be seen, but such declines are unlikely to commence without preventing malaria through impregnated bednets and making RDTs and ACTs widely available and used. We did not have community advocacy campaigns to improve awareness of the necessary care for children, febrile or not. Therefore, the results obtained are solely attributable to the provision of training and commodities to CHWs as close to sick children as possible. As witnessed in this study, coverage increased, showing that when malaria is rapidly diagnosed and effectively treated with the artemisinin derivatives, patient outcomes improve; this also reduces the period during which the infection can be transmitted to others [16].

For many years, there has been an assumption that fevers are the entry point for diagnosis of malaria and that only febrile children come for care. One speaks of malaria as a febrile illness. Afebrile malaria is rarely recorded. CHWs documented the symptoms of the patients who sought their care, and in about 2.7% afebrile cases, the majority with no other symptoms, they judged that an RDT to confirm or rule out malaria was warranted and found that almost half the afebrile patients harbored a *P. falciparum* malaria infection. Our finding that afebrile children came for care and half were parasite positive is unusual. This finding applies mainly to Burkina Faso and Uganda where malaria prevalence was greatest, suggesting that the results on afebrile malaria may be a feature of high transmission in these 2 countries, as in areas with lower prevalence of parasites, *P. falciparum* is more likely to result in symptomatic infections. The study did not test for the presence of HbAS hemoglobinopathies that attenuate clinical malaria [17].

The results reflect the pattern of malaria in children presenting to a CHW in the community. Not all who were ill would have sought care from the CHW. Use of RDTs presents the challenge of what to do with children who test negative for malaria, especially when these children are without danger signs. Some studies have shown that the decline in antimalarial treatment accompanying RDT use has had a corresponding effect on antibiotic use [18]. In this study, RDT negative cases without danger signs were not treated with antibiotics, but were told to return if they did not get better; they were not always followed up at home for outcome. The only patients who were all followed up systematically both at the referral center and at home were those with danger signs eligible for rectal artesunate. Thus the final outcome of uncomplicated cases is not always available.
